# Treatment experience for different risk groups of Kaposiform hemangioendothelioma

**DOI:** 10.3389/fonc.2024.1336763

**Published:** 2024-06-05

**Authors:** Miaomiao Li, Xusheng Wang, Rosalind Kieran, Zheng Wei Sun, Yubin Gong, Hongzhao Lei, Bin Sun, Li Xiao, Yanlin Wang, Song Wang, Zhiyu Li, Luying Wang, Renrong Lv, Feng Xue, Jianfeng Ge, Changxian Dong, Ran Huo

**Affiliations:** ^1^ Department of Burn and Plastic Surgery, Shandong Provincial Hospital, Shandong University, Jinan, Shandong, China; ^2^ Department of Hemangioma and Vascular Malformation Surgery, People’s Hospital of Zhengzhou University, Zhengzhou University, Zhengzhou, Henan, China; ^3^ School of Pharmaceutical Sciences (Shenzhen), Sun Yat-sen University, Shenzhen, Guangdong, China; ^4^ Department of Oncology, Early Cancer Institute, University of Cambridge, Cambridge, United Kingdom; ^5^ Department of Oncology, Cambridge University Hospitals NHS Foundation Trust, Cambridge, United Kingdom; ^6^ Department of Radiology, Guangdong Women and Children’s Hospital, Guangzhou, Guangdong, China; ^7^ Plastic Surgery Hospital, Chinese Academy of Medical Sciences and Peking Union Medical College, Beijing, China

**Keywords:** Kaposiform hemangioendothelioma, Kasabach-Merritt phenomenon (KMP), vascular tumor, sirolimus, coagulation disorder

## Abstract

**Background:**

Kaposiform hemangioendothelioma (KHE) is a rare vascular tumor with a high risk of mortality. Few studies with large samples of KHE have been reported. KHE may develop into the Kasabach–Merritt phenomenon (KMP), which is characterized by thrombocytopenia and consumptive coagulopathy. The features of severe symptomatic anemia and life-threatening low platelets make the management of KHE associated with KMP challenging.

**Objective:**

The aim of this study was to examine the clinical characteristics of patients with KHE and discuss the treatment experience for different risk groups of KHE.

**Methods:**

Through a retrospective review of 70 patients diagnosed with KHE between 2017 and 2022 in our center, we classify lesions into three clinicopathological stages based on the tumor involving depth, and divided the severity of KHE into three levels by estimating clinicopathological stages and severity of thrombocytopenia. Treatments of different severity groups were estimated with sufficient data.

**Results:**

In our cohort, 27% were neonates, and KHE lesion occurred at birth in 84% of patients. There was a slight male predominance (32 girls and 38 boys). Common clinical characteristics included associated coagulation disorder (100%), locally aggressive cutaneous blue–purple mass (89%), thrombocytopenia (78%), and local pain or joint dysfunction (20%). The lower extremities were the dominant location (35%), followed by the trunk (29%), the maxillofacial region and neck (24%), and the upper extremities (10%). Of the total cohort, 78% developed KMP; the median age at which thrombocytopenia occurred was 27.8 days. The median platelet count of patients who were associated with KMP was 24,000/µL in our cohort. Ninety-two percent of patients were given surgery treatment and 89% of these patients were given high-dose methylprednisolone (5-6 mg/kg daily) before surgery. In 55 patients with KMP, 36% were sensitive to high-dose corticosteroid therapy. Patients from the low-risk group (eight cases) underwent operation, all of whom recovered without recurrence after a maximum follow-up of 5 years. Out of 26 patients from the high-risk group, 25 underwent surgery treatment, with 1 case undergoing secondary surgery after recurrence and 1 case taking sirolimus. Out of 36 cases from the extremely high-risk group, 32 underwent surgery (including 2 cases who underwent external carotid artery ligation and catheterization), 3 of whom underwent secondary operation after recurrence, and the remaining 4 cases took medicine. The mean length of having sirolimus was 21 months; two cases stopped taking sirolimus due to severe pneumonia. Two cases died at 1 and 3 months after discharge.

**Conclusions:**

Our study describes the largest assessment of high-risk patients with KHE who have undergone an operation to date, with 5 years of follow-up to track recovery, which provides invaluable knowledge for the future treatment of patients with KHE and KMP from different risk groups: Early surgical intervention may be the most definitive treatment option for most patients with KHE; multimodality treatment is the best choice for the extremely high-risk group.

## Highlights

- **What is already known about this topic?**

Kaposiform hemangioendothelioma (KHE) is a rare vascular tumor with high risk to develop into the life-threatening Kasabach–Merritt phenomenon (KMP), which has no systematic treatment guidelines.

- **What does this study add?**

We retrospectively review KHE paraffin and classify KHE lesions into three clinicopathological stages based on the tumor involving depth.
We divide the severity of KHE into three levels by estimating clinicopathological stages and severity of thrombocytopenia.

- **What are the clinical implications of this work?**

Our work provides an invaluable experience for the treatment of patients with KHE and KMP from different risk groups in the future.

## Introduction

Kaposiform hemangioendothelioma (KHE) is a rare vascular tumor, which typically presents first during the neonatal period or infancy as an enlarging, ill-defined, blue–purple cutaneous mass (1). No accurate prevalence data or incidence was reported, although the estimated prevalence has been reported at 0.91 in 100,000 children in Massachusetts (2). As its name implies, KHE clinically shows aggressive features like Kaposi sarcoma, but without distant metastasis (3). KHE was first named by Zukerberg in 1993 (1) as unknown invasively soft tissue masses presented in nine infancies. It was described as a vascular tumor with locally aggressive lymphangiomatosis and Kasabach–Merritt syndrome (KMS) (4). Zukerberg also reported that the tumor that was due to the Kasabach–Merritt phenomenon (KMP) was KHE instead of hemangioma (1). KHE may infiltrate the dermis, fat, one muscle or multiple muscles, or even bones (5). Multiple-lesion involvement is uncommon and several cases with retroperitoneal or intrathoracic involvement have been reported (6). Cases of other anatomic locations—biopsy-proven hepatic tissue (7), spleen (8, 9), kidney (10), tongue (11, 12), bone (13), and multiple visceral organs (14)—have been reported gradually. KHE may be misdiagnosed due to its rarity and doctors’ poor awareness of this disease. It may be confused with rapidly involuting congenital hemangioma (RICH), which rarely associates with transient thrombocytopenia (15). RICH involutes almost completely rapidly in most cases, whereas lesions of KHE are expansive and aggressive.

KHE notably has a high risk of developing into KMP, a life-threatening and intractable disease with clinical characteristics of KHE or tufted angioma (TA) with thrombocytopenia and coagulopathy (16–18), especially with hypofibrinogenemia, typically complicated by severe anemia, bleeding, and disseminated intravascular coagulation (DIC). KMS was first reported by Kasabach and Merritt in 1940 (19); it was described as a huge hemangioma with thrombocytopenia. Until 1997, Enjolras and Sarkar retrospectively analyzed pathological tissue of KMS and proposed that only KHE and TA with severe thrombocytopenia, secondary fibrinogen reduction, microangiopathic hemolytic anemia, and consumptive coagulopathy could be called KMP instead of KMS (5).

Although a unified definition has been made, there is still a lack of standardized treatment plan for KHE to date. Few studies with large samples of KHE have been reported (2, 20), and most studies are restricted case reports with limited experience of treating KHE (21). Given the challenging management considerations of KHE, this study retrospectively reviews 70 patients diagnosed with KHE over 5 years by analyzing the changes in platelet counts and blood coagulation index during the perioperative period and evaluating the treatment effect within a maximum of 5 years of follow-up, examines the clinical characteristics and complications of patients with KHE, and discusses the management options and outcomes.

## Methods

To form our cohort of patients, we retrospectively reviewed the medical records and laboratory data of 70 patients diagnosed with KHE referred to the Hemangioma and Vascular Malformation Center at Henan Provincial People’s Hospital between 2017 and 2022 using the search engines with terms such as KHE, KHE with thrombocytopenia, KMP, and KMS. All the retrospective review behavior was permitted by our Institutional Review Board. The cohort cases were reconfirmed by more than two physicians and two pathologists with extensive experience based on tumor photographs, medical records, imaging, and pathological results.

All our medical reports and laboratory data were reviewed; platelet counts and coagulation indices were collected to define KMP. Hematoxylin–eosin staining, Immunol staining, operative notes, and intraoperative photographs were retrieved to examine the depth of infiltration for patients who were given surgical treatment. We recorded the tumor depth of patients who were given conservative treatment, by reviewing magnetic resonance imaging (MRI) and color Doppler imaging characteristics.

**Figure 1:**
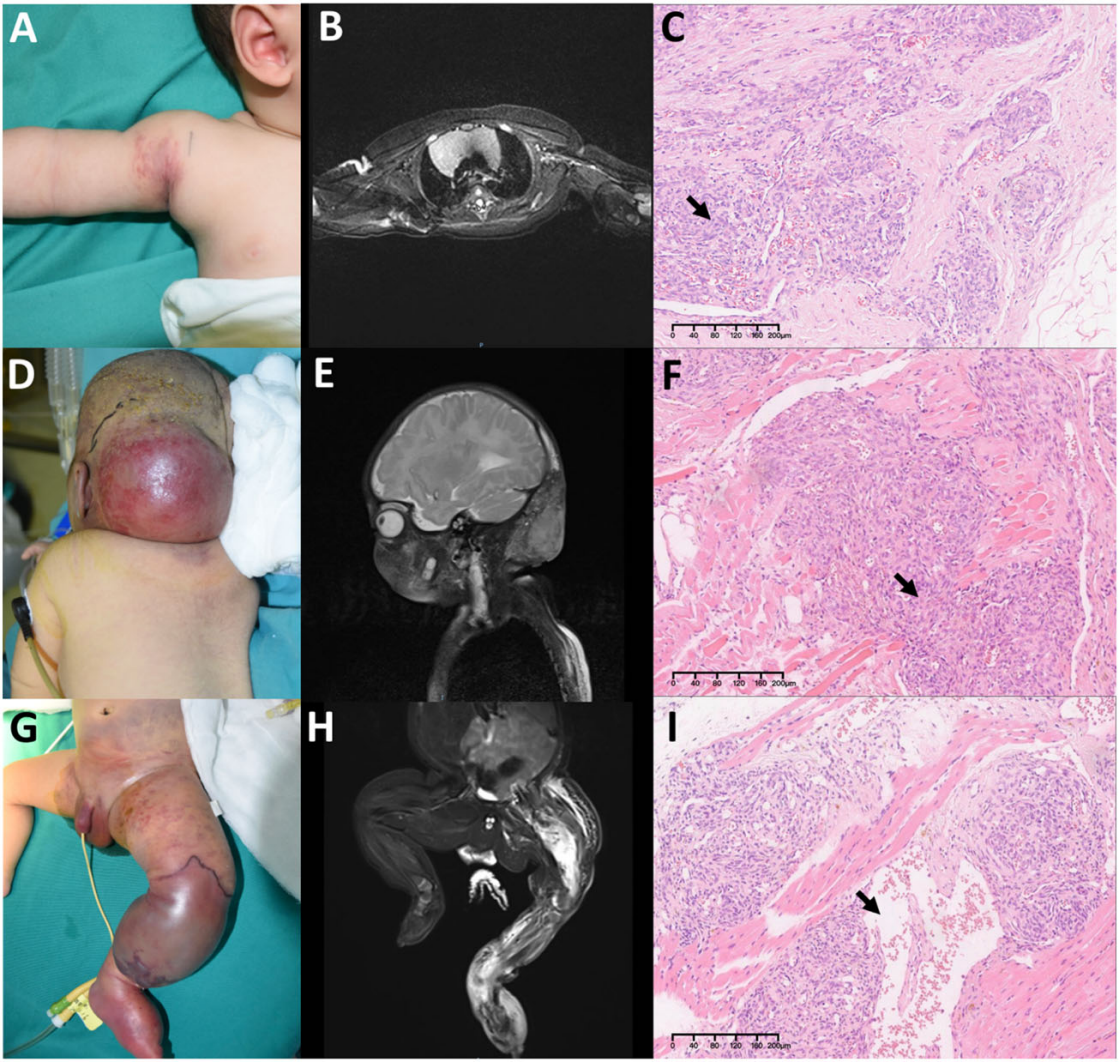
Three clinicopathological stages in Kaposiform hemangioendotheliomas (KHE): **(A–C)** Stage I: A 3-month-old boy diagnosed with KHE on his right armpit **(A)**. The lesion involves the dermis and subcutaneous tissue **(B)**. There are several tumor nodules in the dermis (arrow). Slit-like spaces are also noted **(C)**. **(D–F)** Stage II: A 4-month-old boy diagnosed with KMP on his occiput **(D)**. The lesion involves occiput muscles with ill-defined margins **(E)**. Pathologically confirmed numerous spindle-like neoplastic cells crisscross and infiltrate muscles (arrow) **(F)**. **(G–I)** Stage III: A 23-day-old boy was found to have a congenital red mass on his left leg **(G)**. The lesion involves multiple muscles, the left fibula, and the joint area **(H)**. Plenty of neoplastic cells with some mitoses in an infiltrative, multinodular pattern; lymphatic luminal structures were not rare (arrow) **(I)**. Original magnification for all H&E staining is ×10.

Based on the tumor involving depth, we classify lesions into three clinicopathological stages (see **Table 1**): Stage I (**Figures 1A–C**), Stage II (**Figures 1D–F**), and Stage III (**Figures 1G–I**).

**Table 1:** Three clinicopathological stages according to tumor involving depth.

Clinicopathological stage	Tumor involving depth
**Stage I**	Lesions invade the skin, dermis, and fat
**Stage II**	Lesions invade the skin, dermis, and fat, with deep infiltration in muscles, but do not break through the periosteum
**Stage III**	Lesions invade the articular cavity, retroperitoneum, chest, or pelvis sites, with multiple lesions, or with bone destruction

We divide the severity of KHE into low-risk, high-risk, and extremely high-risk levels by estimating all the above elements (see **Table 2**).

**Table 2:** Three risk levels based on the severity of Kaposiform hemangioendotheliomas (KHEs).

Risk level	Principles of evaluation
**Low risk**	Lesions involving Stage I or Stage II without thrombocytopenia
**High risk**	Lesions involving Stage II or Stage III with thrombocytopenia, platelet count <100×10^3^/µL but ≥30×10^3^/µL
**Extremely high risk**	Lesions involving Stage II or Stage III with platelet count <30×10^3^/µL and the level of platelet count tends to continue to fall

The anatomical stage and severity grade of KHE, which played important roles in selecting remedies, were evaluated according to the above elements. Data of complications before and after hospitalization have been noted as follows: thrombocytopenia was defined as platelet count <100,000 cells/µL; consumptive coagulopathy was defined as hypofibrinogenemia (fibrinogen level <2 g/L), hyper-fibrinogen degradation products (FDPs) >5 mg/mL, and high D-dimer (>0.5 mg/mL); and severe anemia (hemoglobin <60 g/L). Other complications included bleeding, DIC, and motor dysfunction of limb. A maximum 5-year follow-up was used to evaluate treatment effect.

Statistical analyses were performed in GraphPad Prism (version 8). *p*-values lower than 0.05 were considered statistically significant. For platelet counts; D2-dimer, FBG, and FDP analyses; ordinary one-way analysis of variance (ANOVA); and unpaired *t*-test were used.

## Results

### Cohort characteristics

The KHE cohort patients came from 17 provinces and 51 cities. A total of 35 patients were from 20 cities in Henan Province, and the rest came from all over the country, but mainly concentrated in East and Central China. We assume that our data can represent KHE features in a large proportion of East and Central China.

In our cohort (see **Table 3**), the youngest was 1 day old and the oldest was 3 years 8 months old; 27% were neonates and the average age was 112 days old. KHE lesions occurred at birth in 83% of patients. The male-to-female ratio was 1.18:1, which showed a slight male predominance. The mean ages of masses that were found and diagnosed as KHE were 40 days and 96 days, respectively. The median age of initial KHE diagnosis was 96 days, almost fourfold longer than the age of patients with KMP. Seventy-five percent of patients were given one single remedy or combined therapies in other hospitals before coming to our center. Treatments ranged from corticosteroids (23 cases), propranolol (11 cases), sirolimus (9 cases), platelet transfusion (7 cases), immunoglobulin (6 cases), vincristine (4 cases), and aspirin (1 case); other treatments included anti-inflammatory treatment, interventional therapy, hemostatic therapy, and cryoprecipitate treatment.

**Table 3:** Baseline characteristics of patients with Kaposiform hemangioendotheliomas (KHEs).

Sex, *n* (%)	
Male	38 (54.3)
Female	32 (45.7)
Age distribution	
0–6 months	62 (88.5)
7–12 months	7 (10)
>13 months	1 (1.5)
Age of KHE accuracy (%)	
At birth	59 (84.2)
After birth	11 (15.8)
KHE clinical characteristics (%)	
Cutaneous blue–purple mass	62 (88.5)
Thrombocytopenia	55 (78.5)
Coagulation disorder	70 (100.0)
Local pain or joint dysfunction	14 (20.0)
KHE location (%)	
Lower extremities	25 (35.7)
Upper extremities	7 (10.0)
Trunk	20 (28.5)
Maxillofacial region and neck	17 (24.2)
Abdominal and pelvic	1 (1.4)
Pleura or intracalvarium	6 (8.6)
Clinicopathological stage	
Stage I	11 (15.8)
Stage II	25 (35.7)
Stage III	34 (48.5)
Risk level	
Low risk	8 (11.4)
High risk	26 (37.1)
Extremely High risk	36 (51.5)
Treatment (%)	
High-dose methylprednisolone	62 (88.5)
Sirolimus	5 (7.1)
Surgery	65 (92.8)

### Tumor characteristics

Common clinical characteristics included a locally aggressive cutaneous blue–purple mass that occurred in 64 cases (91%). The lower extremities were the dominant location (35%), followed by the trunk (29%), the maxillofacial region and neck (24%), and the upper extremities (10%). In our cohort (see **Table 3**), 11 cases (15%) had lesions that invaded the skin, dermis, and fat, with one invading the superficial muscle. Fifty-nine cases (85%) had lesions deep into multiple muscles. Examination of MRI in 24 cases (34%) showed bone destruction: ribs were the most frequently invaded bone, with a proportion of 33%, which was twice as much as lower-limb bones. Surgery revealed that 22 cases had nerve involvement, with the facial nerve, cervical plexus, and brachial plexus being the most common nerves invaded.

Among the 32 extremity cases, 14 (44%) had one or two joint involvement: 4 cases had knee cavity invasion, 2 cases involved shoulder joint cavity, and 1 case had elbow joint destroyed. Of these cases, 28% had local motor dysfunction. Parietal pleura was the most common extracutaneous site: five cases had parietal pleura invasion, with the mediastinum invaded in two cases. Two cases with abdominal and pelvic invasion had complications of pelvic effusion and hydronephrosis, respectively; five cases involved pleural and spinal infiltration; two cases with lesions involved inner ear infiltration, resulting in slight hearing impairment; and one case involved intracranial infiltration, wherein the tumor was found to be connected to the intracranial vein.

Seventy-eight percent of our cohort patients developed KMP. The median time at which thrombocytopenia occurred was 27.8 days, with six cases diagnosed with KMP at the first day after birth. The median platelet count of patients at the initial presentation of KMP was 38,200/µL. All patients with KHE had coagulation dysfunction. Other complications included anemia, which occurred in 23 cases (32.8%), hemorrhage, severe infection, and hydronephrosis.

According to the above anatomical stage, 11 cases were assigned to Stage I in which 2 cases (18%) developed into KMP, 25 cases were assigned to Stage II in which 21 cases (84%) developed into KMP, and 34 cases were assigned to Stage III in which 25 cases (74%) developed into KMP. According to the above severity grade, 8 cases (11%) were in the low-risk group, 26 cases (37%) were in the high-risk group, and 36 cases (52%) were in the extremely high-risk group.

### Treatment

**Figure 2:**
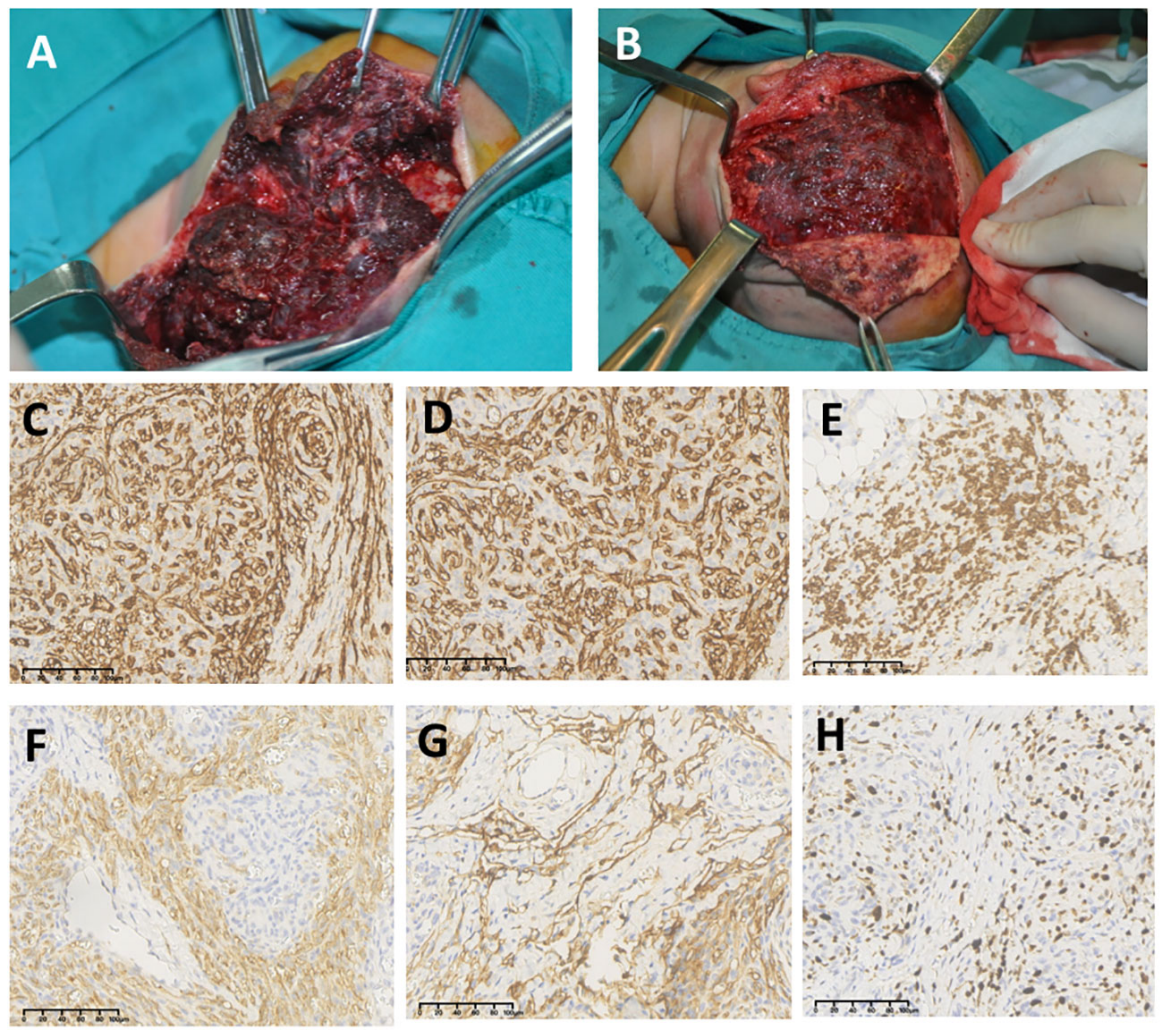
Photographs of intraoperation for nidus of Kaposiform hemangioendotheliomas (KHEs) with the Kasabach–Merritt phenomenon (KMP) **(A, B)**; numerous dark purple thrombi can be seen in the fragile tumor tissue. Immunohistochemistry staining (×20) for KHE tissue on CD31 **(C)**, CD34 **(D)**, GLUT-1 **(E)**, D2-40 **(F)**, LYVE1 **(G)**, and KI67 **(H)**.

A total of 65 (93%) patients were given surgery treatment: 2 of them were given catheterization of external carotid artery and drug perfusion in maxillofacial lesions. **Figure 2** shows typical photographs of intraoperation for nidus of KHEs with KMP. Numerous dark purple thrombi can be seen in the fragile tumor tissue; KHE tissue is positive for CD31, CD34, D2-40, LYVE1, and KI67 on endothelial cells, and GLUT-1 is positive for red blood cells but negative for endothelial cells (**Figures 2C–H**). A total of 63 cases were given partial or total tumor resection, and lesions of 54 cases were totally removed in the first operation. Six (9%) cases recurred after a few months with small lesions, four of whom had thrombocytopenia and were given surgery in the second hospitalization, and two cases had normal platelet counts with no special symptoms and just continued follow-up in an outpatient clinic. Three out of 63 cases had partial surgery, all of whom were given medical treatment during the second hospitalization, and one case was operated on to remove the remaining lesions after 6 months. Of these 70 cases, 5 underwent medical treatment with or without sclerotherapy; all of them were alive with tumors, and 2 cases were still taking sirolimus by the end of follow-up. The mean length of having sirolimus was 21 months; two cases stopped taking sirolimus due to severe pneumonia.

15 KHE without KMP cases were given surgery treatment. All KMP cases were given high-dose methylprednisolone (5-6 mg/kg daily), hemostat, or human immunoglobulin (pH 4) pre-operatively. Thirty-two (58%) cases were sensitive to high-dose corticosteroid therapy. Platelet transfusion was only performed the day before surgery for cases that were not sensitive to corticosteroid or sirolimus. Platelet transfusion alone was not effective for KMP cases as platelet counts dropped rapidly to previous levels within 48 h.

**Figure 3:**
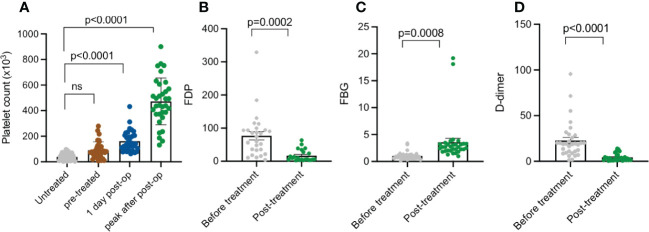
Platelet count, FDP, FBG, and D-dimer summary of patients with KMP at different treatment points. **(A)** Platelet count of untreated patients with KMP, pre-treatment 1 day post-operation, and peak value post-operation. **(B)** FDP of patients with KMP before and after treatment. **(C)** FBG of patients with KMP before and after treatment. **(D)** D-dimer of patients with KMP before and after treatment. 'ns' mean 'no significance'.

Platelet counts and coagulation function indices of KMP cases in the perioperative period were recorded to examine curative effects. The average platelet counts from initial hospitalization was 38.4 × 10^3^ cells/µL, which increased to 97.7 × 10^3^ cells/µL after pre-treatment before operations. One day post-operatively, the platelet counts of these patients significantly rose to 160.9 × 10^3^ cells/µL and later reached a peak value of approximately 462.1 × 10^3^ cells/µL on average (**Figure 3A**). Coagulation function index, including fibrinogen degradation products (FDPs), fibrinogen (FBG), and D-dimer, compared with preoperative values, improved significantly post-operatively (**Figures 3B–D**, *p* < 0.05).

**Figure 4:**
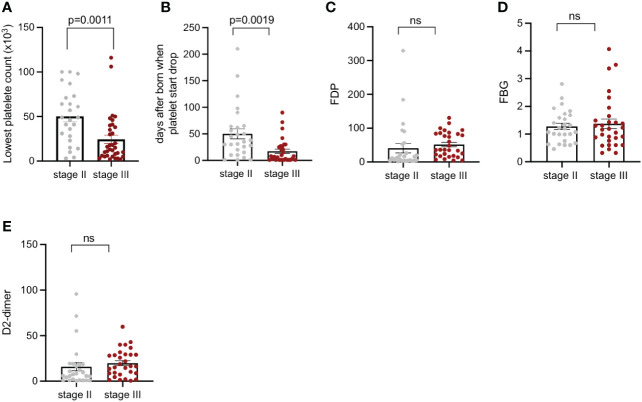
Platelet count, days after being born when platelets start to drop; FDP, FBG, and D-dimer of patients with KMP from Stage II and Stage III. **(A)** Lowest recorded platelet counts of patients with KMP from Stage II and Stage III. **(B)** Days after being born when platelets start to drop from Stage II and Stage III. **(C)** FDP of patients with KMP from Stage II and Stage III. **(D)** FBG of patients with KMP from Stage II and Stage III. **(E)** D-dimer of patients with KMP from Stage II and Stage III. 'ns' mean 'no significance'.

According to the anatomical depth, all KMP lesions were divided into Stage II and Stage III. The lowest platelet counts and coagulation function indices of each stage were recorded, in order to explore whether the anatomical depth had an impact on the severity of KMP. Before surgery, the mean lowest platelet counts of Stage III were 17.5 × 10^3^ cells/µL, which was significantly decreased compared to that of Stage II, which was 50.2 × 10^3^ cells/µL (*p* = 0.0011) (**Figure 4A**). The age when platelet count started to drop for the two stages was 50 days and 17.1 days, respectively (*p* < 0.05) (**Figure 4B**), which was a statistically significant difference, revealing that the anatomical depth had a considerable impact on the age when platelets started to drop. The coagulation function indices, such as FDP, FBG, and D-dimer values, of the two stages had no difference (*p* < 0.05) (**Figures 4C–E**).

In our cohort, 68 of the 70 patients continued to be followed up, and the mean length of tracking time was 2.3 years. Two KMP cases died at 1 and 3 months after discharge, but the immediate causes of death were unknown. Lesions of these two cases were from the chest and back with a large invasive area, deep into the intercostal space, pleura, and brachial plexus. Of the 65 cases who underwent surgery, 2 cases were given sirolimus after operations. They had all stopped taking medicine by the end of the follow-up.

**Figure 5:**
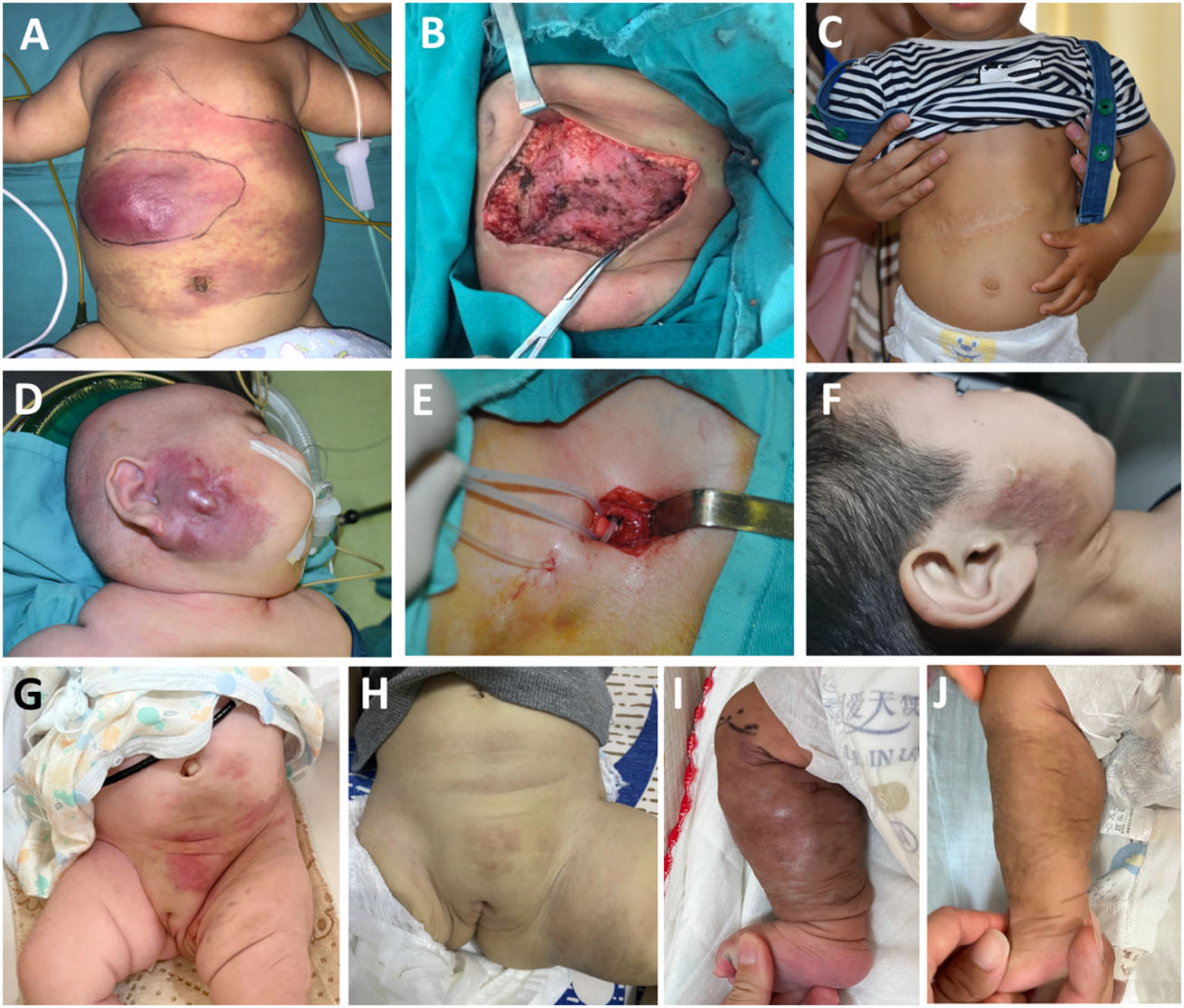
Individualized comprehensive treatment for patients with Kaposiform hemangioendothelioma at different degrees/stages: a 115-day-old boy with enlarging pink mass before operation, after operation, and after 5 years of follow-up **(A–C)**. An 8-month-old boy diagnosed with KMP **(D)**, then with external carotid artery ligation treatment **(E)**; sirolimus was taken afterwards with 5 years of follow-up **(F)**. A 2-day-old girl associated with thrombocytopenia and consumptive coagulopathy **(G)**; methylprednisolone and sirolimus were used with 1-year follow-up **(H)**. A one-day-old boy diagnosed with KMP with a lesion invading almost the entire circumference of the right calf **(I)**. Sirolimus was taken after 1-year follow-up **(J)**. Related MRI scans before and after treatment can be found in supplementary documents.

For most Stage II and Stage III patients, operation alone can completely remove lesions. In **Figure 5**, we show some individualized comprehensive treatments for patients with KMP at different degrees/stages.

Case 1 was a 115-day-old boy with a rapidly enlarging pink mass on the chest wall. The mass became progressively more indurate and purpuric and his platelet count dropped to 13×10^9^ cells/L (**Figure 5A**). He was insensitive to methylprednisolone. After transfusing platelets, he immediately underwent surgery. Post-operatively, his platelet count returned to >100×10^9^ cells/L and his coagulation function returned to normal after 1 month. In the nearly 5-year follow-up, his mass did not recur (**Figures 5B,C**).

For some patients, the lesion might occur in a vulnerable area and therefore surgical removal of the tumor might need to be avoided. For example, case 2 is an 8-month-old boy who was diagnosed with KMP on his right face 3 months after birth. He had been taking methylprednisolone for 5 months in another hospital. The platelet count remained normal, but the size of the mass in the maxillofacial region continued to increase (**Figure 5D**). He underwent external carotid artery ligation and catheterization; 40% urea was injected for 5 days post-operatively; this approach did not efficiently inhibit tumor growth (**Figure 5E**). Finally, sirolimus was given at a dose of 0.8 mg/kg/day and with a weaning dose of methylprednisolone on discharge. Sirolimus was used for 1.5 years. After the 5-year-follow-up, the mass gradually shrank and the platelet count and coagulation function recovered. The only sign of treatment was pigmentation and skin hardening left in the right maxillofacial region (**Figure 5F**).

For some extreme cases in Stage III, drug treatment alone is the usual approach for KMP therapy. For example, in **Figure 5G**, a 2-day-old girl was found to have a hard pink mass in her perineum and left inner thigh at birth. This was associated with thrombocytopenia and consumptive coagulopathy. MRI showed that the lesion was already invading the pelvic cavity; thus, an operative approach was not deemed suitable for the patient. Instead, the patient was treated with methylprednisolone to allow the platelet to increase to normal levels and then sirolimus was used for 10 months. Finally, the pink mass faded away after 1-year follow-up (**Figure 5H**). Another case involved a 1-day-old boy, whose MRI showed a lesion that invaded almost the entire circumference of his right calf. He was diagnosed with KMP and treated with sirolimus (**Figure 5I**). After 1-year follow up, the mass had shrunk significantly, coagulation function had improved, and platelet count had increased (**Figure 5J**).

## Discussion

Since KHE was initially described in 1993, there has been a lack of large-sample retrospective analyses of its features and treatments due to its rarity. Systematic treatment effect analysis and long-term follow-up have played an important role in raising awareness for KHE and KMP. Our study provides a large cohort of KHE clinic features and therapeutic efficacy evaluations.

Most KHE lesions present at birth as locally aggressive cutaneous masses; another quarter were found before the first year of life. Our data showed an unequal sex ratio with a slight tendency for male predominance. Almost half of KHE occurred in extremities, among which one-third had local motor dysfunction during initial hospitalization. On average, patients were diagnosed with KHE 56 days after masses were found. However, patients with KMP were diagnosed and treated much earlier than those with KHE. Over three-quarters of KHE developed into KMP, over one-third of cases showed bone destruction, and all patients with KHE whose lesions involved the parietal pleura, mediastinum, and abdominal and pelvic regions progressed into having KMP.

KHE lesions anatomically involve a spectrum from superficial to infiltrative involving multiple or extracutaneous sites. Our study shows that the vast majority of KMP cases involved deep muscles; however, to date, no criteria have been proposed to risk-classify patients with KHE or KMP based on depth of involvement. Some proposed grading KHE based on its size, but we found that most tumors are complicated by thrombocytopenia, leading to plaques, edema, and inflammation, making it difficult to accurately measure tumor size in both clinical and imaging studies. We proposed using the depth of tumor invasion to grade KHE, which is more objective in reflecting the risk of KHE. Our data suggest that the anatomical depth of patients with KMP is an essential factor that impacts platelet count and time to when platelets start to drop. Lesions invading the parietal pleura, chest, abdomen, retroperitoneum, or mediastinum have been confirmed to be at a high risk of developing into KMP. We also have proven that the deeper the lesions invaded, the earlier thrombocytopenia occurred. However, depth is not the only factor that estimates the severity of coagulation function. Considering that most KHE developed into KMP, we cannot justify its severity only by the classification of invasive depth. The age of patients and complications including thrombocytopenia, consumptive coagulation, and bleeding should also be considered.

Thrombocytopenia and consumptive coagulopathy are the most common complications of KHE (22, 23). Other accompanying symptoms include anemia, hemorrhage, and DIC. Almost all patients with KHE have different degrees of anemia at the time platelet level was dropping (23). However, most patients with KMP had received suspended red blood transfusion at a nearby hospital, which made it difficult for our data to establish the anemia condition accurately. We suggest that KHE severity consists of three levels according to tumor anatomical depth in addition to the state of platelet counts. Extremely high-risk cases have the highest mortality and the highest complication rate as well as irreversible functional damage; in contrast, patients in the low-risk group have minimal complications and the highest cure rate. It has been reported that the bone marrow examination of patients with KMP shows obvious megakaryocyte system maturation disorder, which is manifested by the reduction or even loss of megakaryocytes and platelets in the production plate. However, two patients with KMP in our cohort underwent bone marrow puncture, but no positive results were found. The relationship between bone marrow thrombopoiesis disorder and KHE with thrombocytopenia and coagulation factors should be determined, thus requiring further studies.

Treatment of KHE and KMP remains a challenge due to their rarity. Corticosteroids is the most common medicine that is acutely used to treat KMP (24). Over one-third of patients with KMP were sensitive to methylprednisolone. Sensitivity is defined as platelets gradually rising within 1 week after using corticosteroids (25). The initial dose of methylprednisolone we used was 2 to 3 mg/kg/day. This dose was maintained for less than 1 week. A higher dose (5-6 mg/kg/day) can be used for patients with extremely low platelet count and life-threatening conditions. Rapamycin or sirolimus has been widely used in the treatment of KHE and KMP (26–28). Rapamycin works by inhibiting the expression of various cytokines, including VEGF, and by blocking mammalian target of rapamycin (mTOR) signal pathways, which induces anti-vascular proliferation and promotes apoptosis (29, 30). Vincristine, which can inhibit the proliferation of endothelial cells and promote the apoptosis of tumor cells, is always given to patients with KMP who were not responsive to corticosteroids (31–33). It is not used as a routine drug to treat KMP due to its complications, such as myelosuppression to neonates, peripheral neuropathy, constipation, and intestinal obstruction (25).

Most of the above regimens have potential side effects, but such medicines need to be taken for several months or even years. Furthermore, half of our cohort patients were not responsive to corticosteroids. Most patients were sensitive to sirolimus, but the lack of treatment standard, immunosuppression, and unknown long-term safety are significant concerns. In our cohort, five KMP cases were given methylprednisolone, and only until platelet counts were raised to normal levels was methylprednisolone gradually withdrawn. At the same time, sirolimus was given at a dose of 0.8 mg/m^2^/day, and the concentration of 8–15 ng/mL was maintained. Two patients with maxillofacial KMP were given sirolimus after catheterization of the external carotid artery and drug perfusion. Three cases who had partial surgery were given sirolimus due to stubborn thrombocytopenia.

Early surgical intervention may be the most definitive treatment option for most patients with KHE. For patients in the low-risk group, surgical treatment has the advantage of resecting lesions quickly and directly, usually reducing the risk of thrombocytopenia and also avoiding the need to take long-term toxic medicines. Patients in the high-risk group are classified into three groups: the first group has a stable platelet count and circumscribed lesions; the second group has lesions in a large area, thus affecting appearance; and the last group has lesions affecting function. We recommend complete surgical excision once platelet counts rise for the first group, while multimodality treatment is a contingency plan for the second group, due to the probability of the condition progressing to the extremely high-risk stage at any time; for the third group, we found that early surgery intervention can reduce further functional damage, and of course, it can also be combined with medication treatment.

Rapid recognition and diagnosis are essential for the most vulnerable extremely high-risk group, which has the highest mortality rate. High-dose corticosteroids, hemostat, and human immunoglobulin (pH 4) should be used in life-threatening situations, as well as infusion of suspended red blood cells and fresh frozen plasma with cold precipitation to correct anemia and improve coagulation, respectively. Platelet transfusion should only be performed the day before surgery. Platelet transfusion alone is not effective for patients with KMP, as platelet counts drop rapidly to previous levels within 48 h. Lesions of limited size in the trunk and limbs are recommended to be partially or completely excised after patients reach a stable condition. Multimodality treatment is the best choice for lesions with a large invasive area or those found deep in the intercostal space, pleura, and brachial plexus. Sirolimus is the preferred treatment to increase platelet count; most patients can achieve positive outcomes as a result of this treatment (**Table 4**).

**Table 4:** Treatment recommendations and outcomes for three risk levels of Kaposiform hemangioendotheliomas (KHEs).

Risk group	Treatment	Outcomes
**Low risk** Stage I or II, platelet count ≥100×10³/µL	Surgical treatment is the first choice	8 cases underwent operations, all of them recovered without recurrence after a maximum follow-up of 5 years
	Lesions that cannot be surgically removed: Local injection therapy	
	Corticosteroids, sirolimus	
	Medicine for elevating platelet count: Corticosteroids, pH 4	
**High risk** Stage II or III, 30×10³/μL < platelet count < 100×10³/μL	Correct anemia and improve coagulation function	
	Lesions circumscribed: early surgery intervention	25 out of 26 patients underwent surgery treatment after multimodality treatment with 1 case recurrence; 1 case took medicine for more than 2 years
	Lesions with large area or affecting appearance: Medicine or with interventional medicine	
	Lesions affecting function: early surgery intervention combined with taking medicine	
	Medicine for first aid: corticosteroids, rapamycin, or sirolimus, pH 4	32 out of 36 cases underwent surgery after multimodality treatment, where 3 cases underwent secondary operation after recurrence, 4 cases took medicine, and 2 cases died at 1 and 3 months after discharge.
	Correct anemia and improve coagulation function	
**Extremely high risk** Stage II or III, platelet count < 30×10^3^/μL	Lesions circumscribed: early surgical intervention	
	Lesions with large area or internal organs invasion or maxillofacial; medicine or with interventional medicine	

This study describes the largest assessment of patients with KHE who have undergone an operation to date. Most patients with KHE experience KMP during their disease course. Patients who have been treated before enrolling in our cohort usually had invasive masses with stubborn thrombocytopenia and severe consumptive coagulation. It is crucial that an individualized sequential therapy plan should be adopted according to risk stage as well as the lesions’ location, tumor size, and age. Furthermore, our data and treatment experiences have provided useful information as they have confirmed that our therapy plans are reliable and effective after a follow-up of up to 5 years. This study is the first to propose the concept of clinicopathological stage and severity level of KHE, which can evaluate the severity of KHE to a certain extent and has certain significance for subsequent treatment (**Table 4**).

In summary, our study describes the largest assessment of high-risk patients with KHE who have undergone an operation to date with 5 years of follow-up to track recovery, which provides invaluable knowledge for the treatment of patients with KHE and KMP from different risk groups in the future.

## Data availability statement

The raw data supporting the conclusions of this article will be made available by the authors, without undue reservation.

## Ethics statement

The studies involving humans were approved by Institutional Review Board (IRB) of People’s Hospital of Zhengzhou University. The studies were conducted in accordance with the local legislation and institutional requirements. Written informed consent for participation in this study was provided by the participants’ legal guardians/next of kin. Written informed consent was obtained from the minor(s)’ legal guardian/next of kin for the publication of any potentially identifiable images or data included in this article.

## Author contributions

ML: Conceptualization, Formal analysis, Funding acquisition, Methodology, Project administration, Writing – original draft, Resources, Writing – review & editing. XW: Conceptualization, Writing – review & editing. RK: Writing – review & editing. ZS: Writing – review & editing. YG: Writing – review & editing. HL: Writing – review & editing. BS: Writing – review & editing. LX: Writing – review & editing. YW: Writing – review & editing. SW: Writing – review & editing. ZL: Writing – review & editing. LW: Writing – review & editing. RL: Writing – review & editing. FX: Writing – review & editing. JG: Conceptualization, Data curation, Funding acquisition, Methodology, Supervision, Visualization, Writing – review & editing. CD: Conceptualization, Funding acquisition, Writing – review & editing. RH: Conceptualization, Funding acquisition, Supervision, Writing – review & editing.

## Supplementary material

Supplementary Figure 1 MRI scan before and after treatment for patients in **Figures 5A-C**.

Supplementary Figure 2 MRI scan before and after treatment for patients in **Figures 5D-F**.

Supplementary Figure 3 Ultrasound scan before and after treatment for patients in **Figures 5H, H**.

Supplementary Figure 4 MRI scan before and after treatment for patients in **Figures 5I, J**.
