# Supplementary figures and images for: Treatment experience for different risk groups of Kaposiform hemangioendothelioma
Source: Front Oncol. 2024 Jun 5;14:1336763. doi: 10.3389/fonc.2024.1336763 (PMC11188338; doi:10.3389/fonc.2024.1336763)

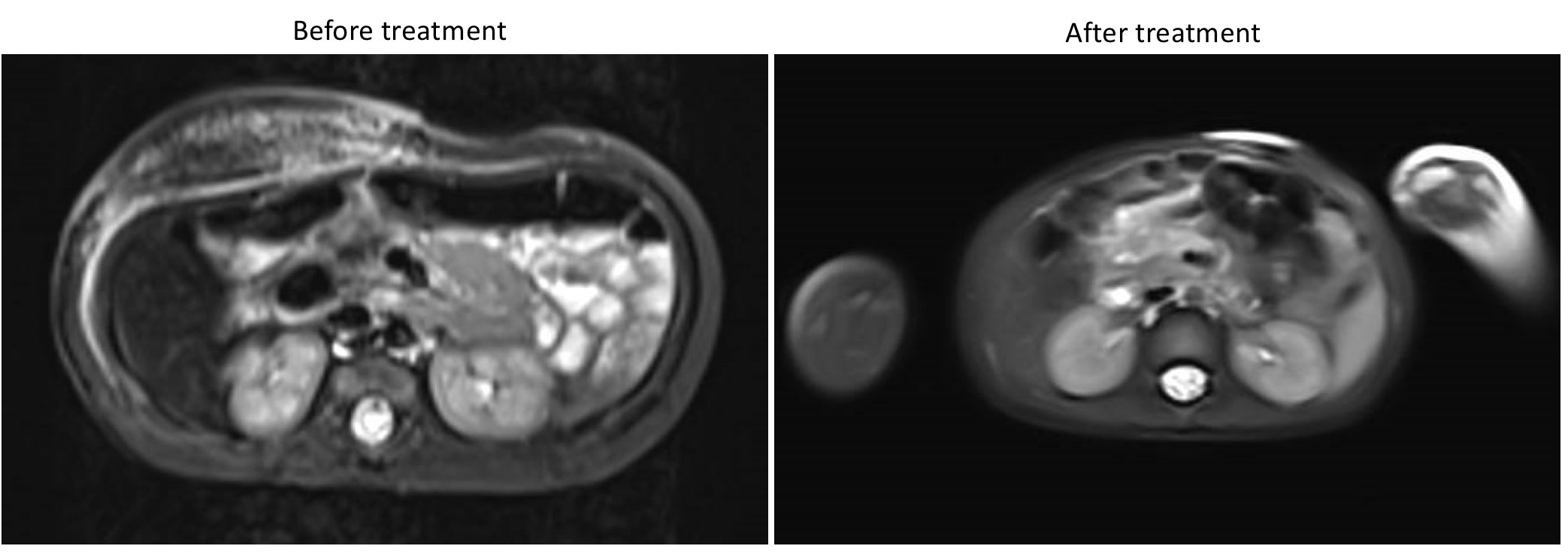

Supplement: Supplementary Figure 1 — MRI scan before and after treatment for patients in Figures 5A-C . [file Image_1.png]

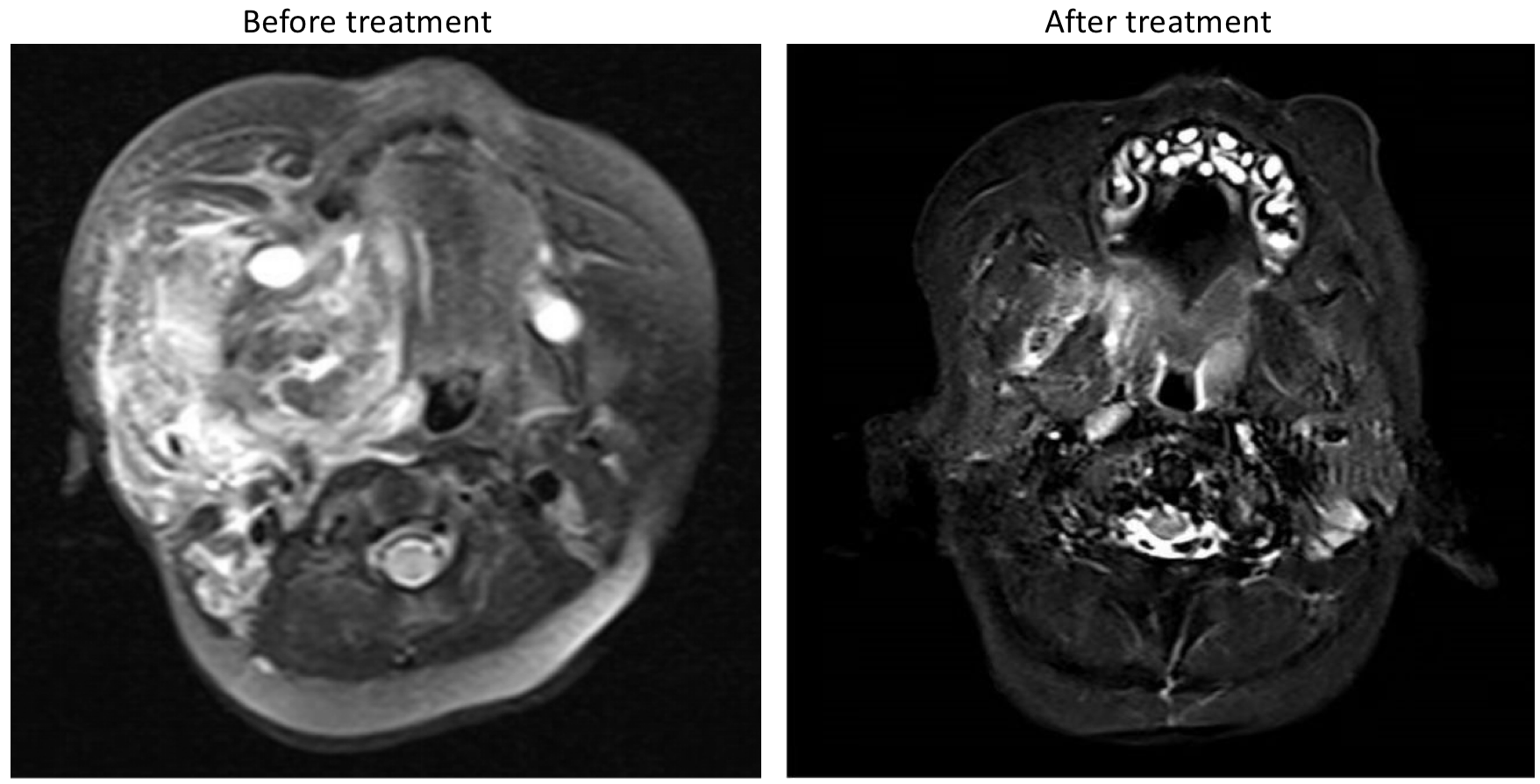

Supplement: Supplementary Figure 2 — MRI scan before and after treatment for patients in Figures 5D-F . [file Image_2.png]

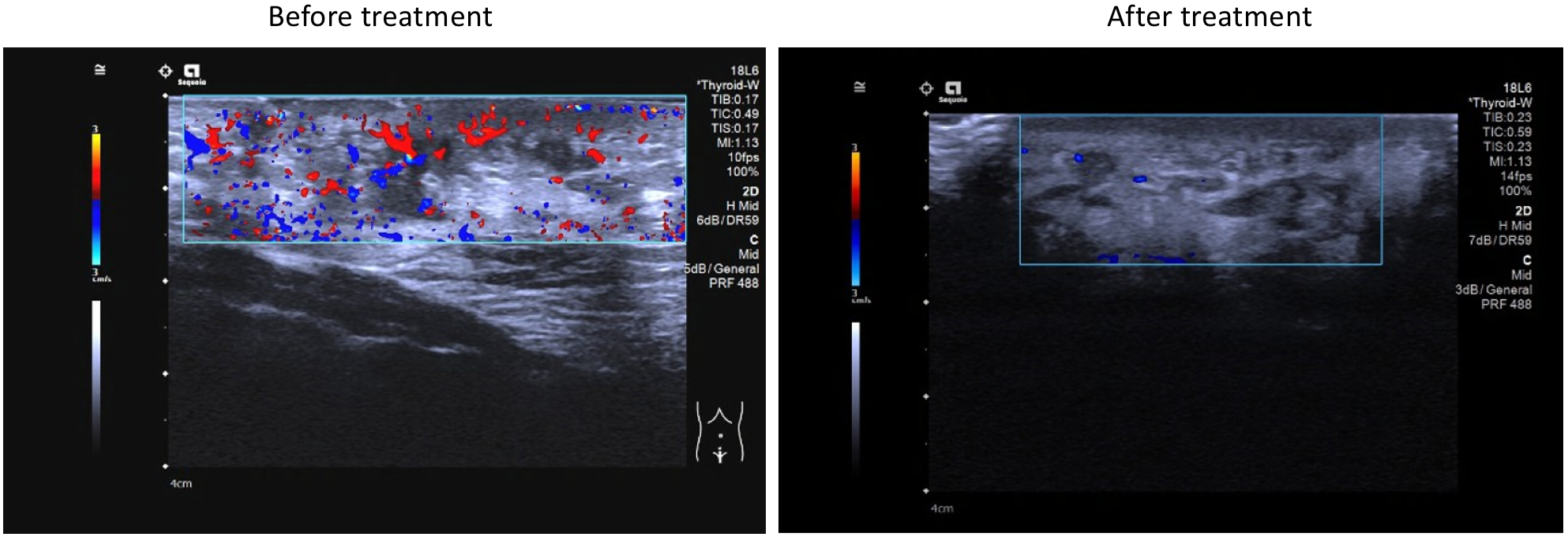

Supplement: Supplementary Figure 3 — Ultrasound scan before and after treatment for patients in Figures 5H, H . [file Image_3.png]

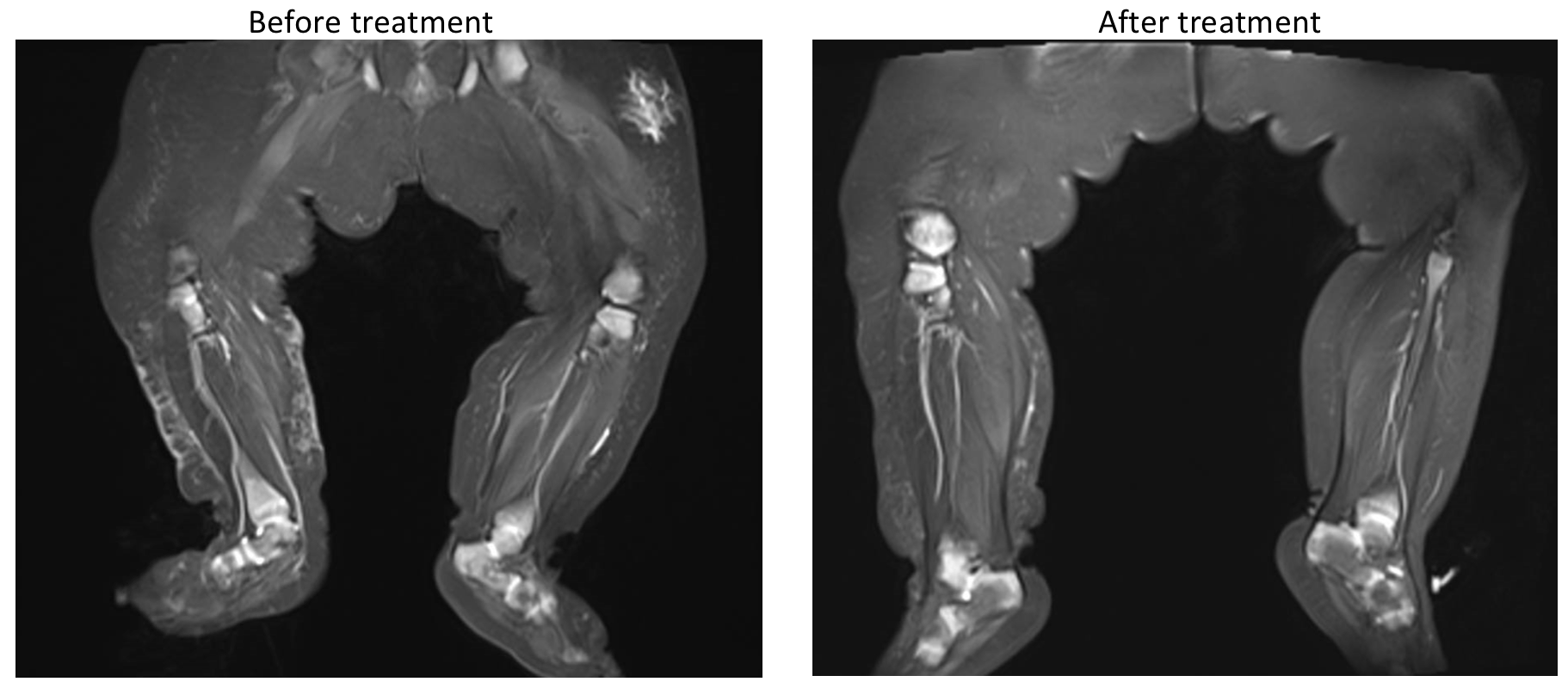

Supplement: Supplementary Figure 4 — MRI scan before and after treatment for patients in Figures 5I, J . [file Image_4.png]
